# Exploring Sound Emission of the Lizard *Pristidactylus valeriae*

**DOI:** 10.3390/ani13243813

**Published:** 2023-12-11

**Authors:** Sebastián Díaz, Antonieta Labra

**Affiliations:** 1 Laboratorio de Ecología Geográfica, Facultad de Ciencias, Universidad de Chile, Santiago 7800003, Chile; sbdiazc@gmail.com; 2 Center for Ecological and Evolutionary Synthesis (CEES), Department of Biosciences, University of Oslo, P.O. Box 1066 Blindern, NO-0316 Oslo, Norway

**Keywords:** distress call, hissing, grunter, spectro-temporal variables, Chile

## Abstract

**Simple Summary:**

Lizards are considered voiceless animals, except for geckos. Nevertheless, increasing evidence shows that different non-gecko lizards emit oral sounds. For example, *Pristidactylus* lizards are known as ‘grunters’ since individuals emit oral sounds when they are under predation risk. We analyzed the characteristics of the sounds emitted by *P. valeriae* when individuals were threatened or captured by a predator. Lizards hissed in both conditions, although hisses were longer when animals were threatened than when captured. The hissing was accompanied by aggressive postures, such as bite attempts. Considering that in Leiosauridae, the family to which *Pristidactylus* belong, oral sound production is present in four out of five genera, this display could be an ancestral character for the group.

**Abstract:**

Lizards, except geckos, are generally considered voiceless organisms, although some species emit oral sounds. For most of these “vocal lizards”, however, there is almost no information on the characteristics of the sounds, precluding exploration of the functionality and evolution of the sounds. *Pristidactylus* are known as “grunter lizards” since individuals emit oral sounds under predation risk. We explored the characteristics of the sounds emitted by *P. valeriae*, recording 17 adults and 1 juvenile when they were threatened and captured by a predator. Only adults emitted sounds with open mouths and displayed aggressive postures, e.g., biting attempts. These sounds correspond to hisses, which lack amplitude or frequency modulation. The lizards emitted longer hisses when threatened than when captured by the predator, which may provide honest information on individuals’ ability to escape. In addition, males may experience higher distress during threats since their hisses had higher aggregate entropy than those of the females. Finally, hissing has been documented in four of the five Leiosauridae genera, the family to which *Pristidactylus* belongs, suggesting that sound emission is ancestral to the family.

## 1. Introduction

Oral sound production is a relatively rare behavior in lizards as compared to other vertebrate taxa, such as birds and amphibians, which has led to the perception of lizards as voiceless organisms [1]. The exception, however, is the members of the Gekkota infraorder, which use vocal communication in different social contexts, i.e., advertisement calls that are emitted during territorial displays and/or mate attraction [2]. Nevertheless, for some non-Gekkotan species (hereafter referred to as lizards), there is also evidence of vocalizations during social interactions, specifically in defensive/agonistic contexts, e.g., [3,4,5,6]. Furthermore, there is growing information on species that vocalize under predation risk, i.e., when a predator threatens or captures individuals, e.g., [5,7,8,9,10,11,12]. Among these cases, the vocalizations emitted by individuals of *Liolaemus chiliensis* when caught by a predator [13] have received some attention. These sounds trigger antipredator responses in conspecifics [14,15], and are modulated by information encoded in the vocalizations [16]. 

Globally, the available information on lizard oral sound production indicates, on the one hand, that this behavior may be more common than generally accepted, and second, that it is displayed in defensive contexts, mainly during predation, but also during intraspecific interactions. Previously, Milton and Jenssen [4] proposed that vocalizations produced by *Anolis grahami* during agonistic interactions may derive from vocalizations uttered as part of antipredator responses. Thus, in some lizard species, sounds emitted under predation risk may have been co-opted to be used in socially aggressive contexts. Moreover, conspecifics might benefit by decoding information about these sounds, including about the sender’s body size [13] or fighting abilities [7]. A case of co-opted vocalizations is the male courtship calls of bowerbirds, which may have evolved from aggressive calls [17].

To understand the evolution of lizard oral sound production and test, for example, the co-option hypothesis, it is necessary to gain more information about these sounds, from their characteristics to their function. In fact, for many species, the available information on oral sound production is just anecdotal, e.g., [18], as has been pointed out in, e.g., [12,19]. In addition, some studies that describe these sounds are based on small sample sizes, either because few vocalizations were analyzed or because a small number of individuals were included, e.g., [20,21]. Although these data support the relatively low occurrence of oral sound production in lizards, these findings, in conjunction with growing documentation of sound production in more species, provide an impetus to continue gathering more and better information and build a solid base to tackle evolutionary questions on lizard oral sound production. In this context, we aimed to contribute by characterizing the emissions of *Pristidactylus valeriae* and to explore the evolution of this behavior by comparing our data with what is already available for related taxa. 

Oral sound production in lizards includes vocalizations and hisses. The latter are not considered proper vocalizations because they lack temporal modulation and frequency modulation [10,12]. Vocalizations and hisses have been documented in species from different families scattered across the phylogeny [5,12,22]. In some cases, however, many taxon members (e.g., congeneric species) emit sounds [5,19,22], providing an exciting opportunity to explore the evolution of lizard oral sound emission. Such is the case for *Pristidactylus* lizards, a genus of ten species distributed in the southern cone of South America, with four in Chile and six in Argentina [23]. In Chile, these species are called grunters, as anecdotal evidence indicates that they emit oral sounds when threatened or captured by a predator [18,24]. For one of these four grunter species, *P. volcanensis*, it has been shown directly that hisses are evoked when individuals are threatened by predators [25]. Similar results have been reported for the Argentinean native species, *P. scapulatus* [20]. 

We studied the characteristics of oral sounds emitted by *P. valeriae*, for which Donoso-Barros [18] indicated that captured lizards respond with violent expulsions of air that resemble a hiss. Later, Lamborot and Díaz [26] added that individuals that are approached or molested emit sound. In this context, we recorded and compared the sounds individuals uttered when threatened or captured by a predator. In addition, we aimed to compare these sounds with those uttered by congeneric species and explore the occurrence of sound emission in members of the *Pristidactylus* family, Leiosauridae. 

## 2. Materials and Methods

*Pristidactylus valeriae* is a species with a very restricted distribution in central Chile [24], inhabiting *Nothofagus* and sclerophyllous forests [18,27]. We conducted the study in San Juan de Piche Nature Sanctuary (33°55.01′ S, 71°03.23′ W, 800 m asl), Alhué, Metropolitan Region, Chile. The site was visited twice during the summer, between the second half of December 2020 and the first half of January 2021. We searched for lizards between 08:00 and 21:00 h. Using noosing pools, we collected 17 adults (10 ♀, 7 ♂) and 1 juvenile of undetermined sex, and their collecting points were geo-referenced. Lizards were kept individually in a numbered cloth bag to be identified and transported to a facility less than an hour’s walking distance from the study site to record their sound emissions. 

Before the trials, lizards remained in their bags in an isolated room maintained at a temperature between 26–28 °C, the field body temperature of the species [28]. Sound recordings were performed between 14:00 h and 18:30 h on the capture day, using a MXL 990 condenser microphone (Frequency Response: 20 Hz–20 kHz) connected to a M-Audio Mtrack 2 × 2 audio interface, which was finally connected to a personal computer. Records were made with the software Ableton Live. 

Each lizard was exposed separately to two treatments: simulating threat and capture by a predator. Although under natural conditions, animals are usually first exposed to the threat by a predator before they may be captured by it, the experimental lizards were first exposed to capture. The capture protocol has previously proved to be successful in obtaining vocalizations from different lizard species, e.g., *Psammodromus algirus*, *Tropidurus catalanensis* and *Aspidoscelis costatus costatus* [7,8,9] and, thus, we decided to start by the capture treatment to ensure we would obtain sound emissions because the species is generally difficult to find and study [24]. Experiments were performed in a room maintained at 27–28 °C. For the capture treatment, we simulated the catch by a predator, e.g., a person caught a lizard [8,9]. A lizard was removed from its bag, and following the protocol of Labra et al. [13], it was carefully gripped with one hand, attempting to reduce heat transfer, while the lizard’s snout was gently touched with the fingers of the other hand. The stimulation was performed for 2 min, and the microphone was placed 10 cm from the lizard’s snout. 

During the threat treatment, we simulated attacks from an aerial predator, e.g., a person’s hand [25,29] for 2 min. The lizard was placed in an empty plastic container (38 × 29 × 18 cm), and after 10 s, the predator quickly approached the lizard, touching and moving it, mimicking a failed capture. The microphone was 15 cm above the center of the container. Between trials, the container was cleaned with 70% ethylic alcohol to eliminate potential chemical traces that could affect the response of the next individual [30]. 

At the end of each trial, we checked the lizard’s body temperature to ensure that this was within the range of the species field body temperature 26–28 °C [28], thus precluding that variations in body temperature might affect the antipredator responses, e.g., [31]. We used a Cu-constantan thermocouple connected to a digital thermometer (Digi-Sense^®^, Cole Parmer, Illinois, US; ± 0.1 °C). After each recording, the thermocouple was cleaned with 70% ethylic alcohol to avoid possible infections. After the first treatment, lizards remained in their bags for around 1hr in the isolated room described above. Then, at the end of the second treatment, we measured the lizards’ snout-vent lengths with a caliper. After that, individuals remained in their bags in the isolated room until they were returned to their collecting points within 18 h of the capture.

### 2.1. Sound Analyses 

The sounds in .WAV format (48 kHz, 24 bits) were analyzed with Raven Pro 1.6 software, using a short-time Fourier transform series with a Hanning window length of 512 points and 50% overlap. For each sound, we measured seven spectro-temporal variables defined in Table 1.

### 2.2. Statistical Analysis

Each individual was characterized by the average values of the spectro-temporal variables of all sounds emitted in each treatment separately. Preliminary analyses showed that body temperatures were similar across treatments and that this variable did not correlate with any of the spectro-temporal variables measured. Therefore, body temperature was not further considered in the analyses. We examined sexual differences in body size using Student’s *t*-tests. To test whether the treatments (threat vs. capture), the sex of the individuals, and the interaction of these factors modulated the sound spectro-temporal characteristics, we used general linear models (GLM), followed by Fisher LSD tests. We performed two analyses because not all individuals emitted sound in both treatments. In the first one, for repeated measures, data for those individuals that responded to both treatments were analyzed. In the second one, for non-repeated measures, we included data for all lizards that responded but considered records from different individuals for the two treatments. The results are shown as mean ± standard error (SE). Statistical analyses were performed with STATISTICA (StatSoft, Inc. Tulsa, OK, USA, 2001).

## 3. Results

During the trials, lizards had a mean body temperature of 27.76 ± 0.37 °C. Females and males had similar snout-vent lengths (t = 0.7; *p* = 0.49), with a mean of 81.29 ± 1.38 mm (range 70.34–90.69); the snout-vent length of the juvenile was 37.4 mm. Two of the 18 individuals, the juvenile and a female, did not emit sounds in any treatment. In the capture treatment, 15 individuals (8 ♀, 7 ♂) responded, emitting 1 to 7 sounds per individual, and we obtained 35 records in this treatment. Only 9 lizards (5 ♀, 4 ♂) emitted sounds during the threat treatment, producing between 1 to 19 sounds per individual, and a total of 65 records were obtained in this treatment. During both treatments, lizards attempted to bite. In addition, during the threat treatment, they tried to escape, although some confronted the predator, inflating their bodies while emitting sounds and occasionally displaying tail-wave. Only 8 individuals (4 ♀, 4 ♂) emitted sounds in both treatments. 

**Figure 1:**
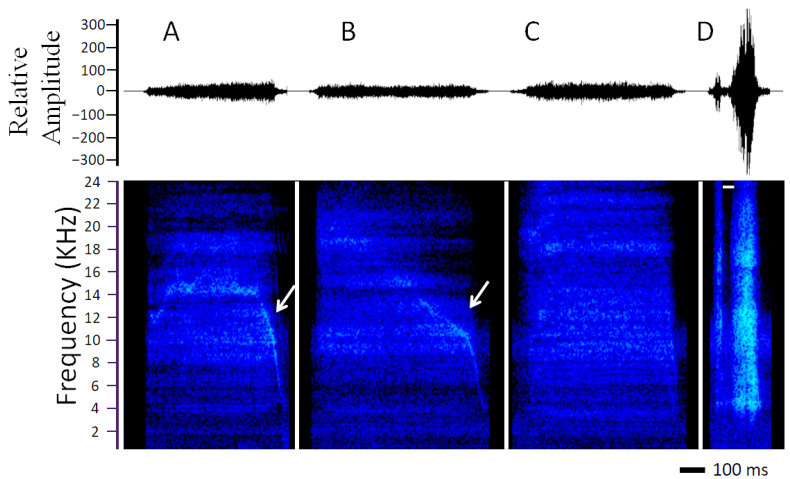
Oscillograms (**top**) and spectrograms (**bottom**) of the two hiss patterns emitted by *Pristidactylus valeriae* under predation risk. (**A**,**B**)—Hisses with modulation indicated by the arrows, emitted by two females. (**C**)—Simple and (**D**)—simple with a silence indicated by the white line. A female (snout-vent length—SVL—83.55 mm) emitted hiss A, and hisses B and C were emitted by another female (SVL = 82.06 mm). These three hisses were uttered during the threat treatment. Hiss D was emitted by a male (SVL = 81.88 mm) during the capture treatment.

Sound emissions occurred during air exhalation with the mouth opened, and these emissions correspond to hisses, i.e., sounds that mainly lacked amplitude and frequency modulation or harmonics, approaching the characteristics of white noise (Figure 1). These hisses covered a broad frequency range, even extending into the ultrasound (>20 kHz). Inspections of the spectrograms revealed two hiss patterns: 1—With modulation (Figure 1A,B). These hisses constituted 15% of all records and included a descending frequency-modulated component. This pattern was only registered during the threat treatment and was only emitted by females, three of the five that hissed. 2—Simple (Figure 1C). These hisses were observed in both treatments and do not show evidence of frequency modulation. They were the most frequent (85%) recorded in both treatments. Some of these simple hisses (N = 6) included a silence with a mean duration of 37 ± 8.95 ms (Figure 1D).

**Figure 2:**
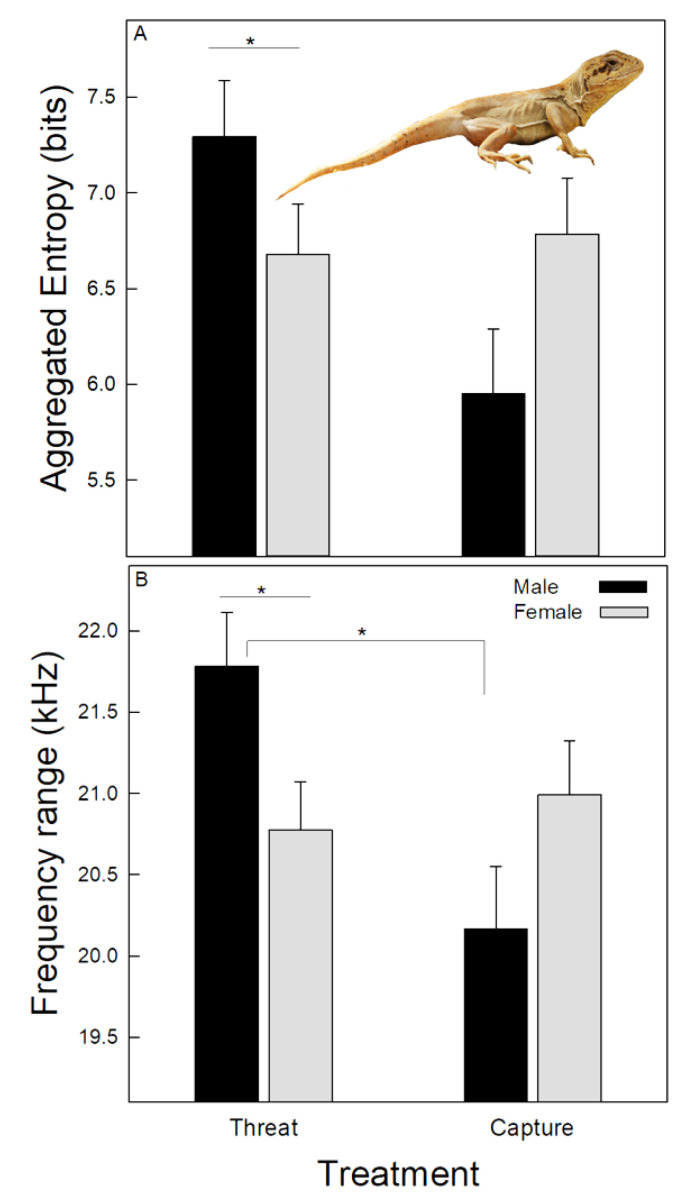
Mean ± standard deviation of spectral variables of the hisses emitted by males and females of *Pristidactylus valeriae* when threatened and captured by a predator. (**A**)—Aggregated entropy. The figure also shows an individual of the species. (**B**)—Frequency range. * = *p* < 0.05.

Table 2 shows the mean values of the seven spectro-temporal variables of hisses recorded in both treatments, pooling the data of the different hiss patterns. Both GLM analyses showed that hiss duration differed significantly between treatments (Table 3), which was longer during the threat than during the capture treatment. In addition, both analyses showed that the low and center frequencies were similar between treatments and sexes. However, the two GLM analyses did not show consistent results for the other four variables. The analyses for repeated measures showed that the high frequency was significantly modulated by sex, which was higher in males than females (23.99 ± 0.01 kHz vs. 23.72 ± 0.1 kHz). On the other hand, the GLM for non-repeated measures showed that the peak frequency was modulated by the treatment, which was higher in the threat than in the capture treatment (Table 3). These analyses also showed that the interaction between treatment and sex modulated the last two variables; in the threat treatment, males hissed with higher aggregated entropy than females (Figure 2A). Finally, when threatened by the predator, males’ hisses showed a broader frequency range than those of females and also compared with hisses that males emitted when being captured (Figure 2B).

## 4. Discussion

Individuals of both sexes of *P. valeriae* hissed with their mouths open when threatened or captured by a predator, accompanied by aggressive behaviors, such as bite attempts. Hisses are not considered proper vocalizations, as they are white noise lacking clear harmonic structures [10,12]. However, some hisses of *P. valeriae* showed a frequency-modulated component, as was recorded in *P. volcanensis* [25]. 

The hisses of *P. valeriae* lack a temporal organization, such as those vocalizations composed of repeated notes at regular intervals emitted by different Gekkota members, e.g., [32,33,34]. On the other hand, the hisses of *P. valeriae* showed high aggregate entropy (high disorder), almost twice that of the values recorded for the vocalizations and hisses of other vertebrates, e.g., Sirenia, Galliformes and Anura [35,36,37], including the vocalizations of the lizard *Aspidoscelis costatus costatus* [9]. In addition, these hisses covered a wide frequency range, from 0.9 kHz to >20 kHz. However, since the microphone was developed to record frequencies up to 20 kHz, frequencies above this limit should be considered cautiously since the microphone’s sensitivity is unclear. In addition, the actual upper limit of the *P. valeriae* hisses needs to be clarified. Future hiss recordings of this species should consider using a microphone that picks up frequencies above 20 kHz. On the other hand, considering the broad frequency range of the recorded hisses, it is likely that *P. valeriae* may respond to some frequency range of these sounds, particularly up to 8 kHz, but probably not above 14 kHz. This proposition is based on the fact that the hearing range (i.e., physiological auditory response) of different lizard species is between 0.1 and 8 kHz [38,39,40], although some few species are sensitive up to 14 kHz [41]. 

For the vast majority of the lizard species that emitted oral sounds, vocalizations, or hisses, there is almost no information on their functionality [10], as is the case of *P. valeriae*. Potentially, these hisses may be eavesdropped on by conspecifics to reduce their own predation risk [14,16], may function as predator deterrents, e.g., [42], or may attract secondary predators [43]. There is no specific information on the predators of *P. valeriae*. However, these likely include raptors such as *Falco sparverious*, a species that commonly preys upon lizards [44], and *Accipiter chilensis*, which preys upon the congeneric species *P. torquatus* [45] and was observed at the study site close to (<100 m) an individual of *P. valeriae*. Other predators may include foxes [46] and snakes [47], such as *Galvarinus chiliensis,* which was observed close to an individual of *P. valeriae* (<50 m). Considering this predatory guild, the frequency range of the hisses of *P. valeriae* overlaps with the hearing sensitivities of raptors [48] and canids [49,50], and thus, potentially, these predators may respond to the *P. valeriae* hisses. In contrast, snakes are sensitive to low-frequency sounds <1 kHz [51], and would be unlikely to respond to these hisses.

*Pristidactylus valeriae* hissed under predation risk, but some sound characteristics were treatment and sex-dependent since; for example, hisses emitted under threat were longer than those emitted under capture. Even if hisses only involve normal respiratory movements [52], prolonged and forced movements might require extra energy. In this context, emitting longer hisses when there is more probability to survive, threat vs. capture, may provide honest information on endurance to fight or escape, e.g., [53] and thus increase the chances to deter the predator. This information may be reinforced by the displays exhibited; some individuals confronted the predator, inflated their bodies, and tail-waved, a pursuit-deterrent behavior [54]. However, we cannot rule out that the tail display may direct attacks on this body part [55]. Analyses of the relation between endurance and hiss duration, or its occurrence, emitted under different interactions with the predator may provide some insights to understand the variations in hiss duration. These analyses can also contribute to unraveling why not all the individuals emit oral sounds, which seems to be a common phenomenon in those lizard species that show this behavior [7,8,9,13].

The interaction between treatment and sex modulated the aggregate entropy, which was higher in males than females when threatened by a predator. This result suggests that males may experience a higher distress in this condition than females. Data show that vocalizations emitted under more stressful conditions have an increase in the occurrence of noise or other nonlinear phenomena, e.g., [56]. This is considered a potential honest signal of fear or arousal of the sender [57], which finally triggers more evocative responses in conspecifics [16,58,59]. There is no information on the social system of *P. valeriae*, and thus, it is unclear why males and not females hissed with higher aggregated entropy while threatened. In addition, male hisses showed a broadening of the frequency range under this experimental condition, which may allow for a more extensive spectrum of species to respond to the hisses, including secondary predators sensitive to high-frequency sounds, such as canids, e.g., [49]. However, since this result is associated with variation in the high frequency, it should be taken cautiously for the reasons previously discussed associated with the microphone characteristics. 

The observed differences between sexes and predatory contexts in the hisses emitted by *P. valeriae* suggest that the sounds may provide information for different receivers, i.e., conspecifics and/or predators [60]. In addition, hisses with a frequency-modulated component in the threat treatment suggest that lizards may have some rudimentary structures that allow for sound modulation [61]. Studies of the larynx anatomy are required to explore this possibility and any potential sexual dimorphism in these structures since this frequency-modulated component was only recorded in females. 

Hisses are sounds with a simple structure and, at least for snakes, data show that they have high similarities across species [52]. In light of this, and considering that vocalizations uttered under predation risk tend to be conservative across taxa, e.g., [62,63], it can be hypothesized that hisses of *Pristidactylus* species may be similar across taxa. Table 4 shows the available information on the hiss characteristics of three species, revealing that the most closely related taxa, *P. valeriae* and *P. volcanensis*, differed in these characteristics and that the hisses of the latter species are more similar in frequency range and duration to those of the Argentine species, *P. scapulatus*. We can rule out that these results are a consequence of adults’ body size differences since both native species from Chile are smaller but similar in size: *P. volcanensis* 84–96 mm [26], *P. valeriae* 70–91 mm (present study), than *P. scapulatus* 105 mm [64]. Habitat structure may modulate these spectro-temporal differences, considering that *P. volcanensis* and *P. scapulatus* inhabit open rocky areas with scrubs [26,64], while *P. valeriae* inhabits more closed habitats, such as *Nothofagus* forests [24]. However, based on the acoustic adaptation hypothesis [65], opposite results would be expected, i.e., lower peak frequencies, longer duration, and a narrower frequency range in closed than open environments. Although some studies support this hypothesis, e.g., [66], others only show partial or no support for it, showcasing the complexity involved in the evolution of vocalizations, e.g., [67,68,69]. In the case of *P. valeriae*, the need for more information on the target audience (i.e., predators/conspecifics) of the hisses precludes providing a plausible hypothesis to explain the observed trends. 

The Leiosauridae family includes 35 species, separated into two subfamilies, Enyaliinae and Leiosaurinae, with *Pristidactylus* belonging to the latter [70]. Data indicate hiss production under predation risk in the three Leiosaurinae genera, in eight of the eighteen species of the subfamily ([11,20,64], present study). In the case of the Enyaliinae, there is a report for only one of the two genera, including one of the 17 species of this subfamily [71]. Altogether, this information suggests that hiss production, further than being present in the ancestor of the Leiosaurinae subfamily, would have been present in the ancestor of the Leiosauridae family. 

## 5. Conclusions

Our study confirms the occurrence of hiss emission by individuals of *P. valeriae* when they were threatened or captured by a predator. Hisses emitted under these two conditions showed some differences, partially modulated by the sex of the individuals. These results suggest that hisses may contain some information, although it is unclear which the target audience is, i.e., conspecifics and/or predators. Considering that hiss production would have been present in the ancestor of the *Pristidactylus* family, Leiosauridae, exploring the function of these hisses in *P. valeriae* and other members of this family may provide relevant information to unravel the evolution of a character, oral sound production, traditionally under-recognized character in lizards.

## Figures and Tables

**Table 1:** Description of acoustic parameters measured in the hisses emitted by individuals of *Pristidactylus valeriae.* s = seconds, kHz = Kilohertz.

Variable	Description
Duration (s)	Time from the beginning to the end of the sound
Low frequency (kHz)	Lower frequency limit of the sound
Center frequency (kHz)	The frequency that divides the sound range frequency into two intervals of equal energy
High frequency (kHz)	The upper-frequency limit of the sound
Peak frequency (kHz)	The frequency at which the maximum power occurs in the sound
Delta frequency (kHz)	Difference between the upper and lower frequency limits of the sound (frequency range)
Aggregate entropy (bits)	Measurement of the disorder of the sound, which analyzes the energy distribution in the sound

**Table 2:** Mean ± standard errors (minimum–maximum values) of the spectro-temporal variables of the hisses recorded in *Pristidactylus valeriae* in two experimental conditions, i.e., capture and threat by a predator. Data for the simple and with modulation patterns were pooled. *n* = number of individuals that hissed in each treatment.

	Capture (*n* = 15)	Threat (*n* = 9)
Duration (ms)	124.85 ± 16.22 (52–268)	265.01 ± 46.00 (51.2–434.14)
Low Frequency (kHz)	3.13 ± 0.27 (0.90–4.55)	2.69 ± 0.24 (1.70–4.16)
Center frequency (kHz)	9.06 ± 0.59 (4.88–13.22)	9.67 ± 0.32 (7.97–10.76)
High frequency (kHz)	23.72 ± 0.18 (21.43–24.0)	23.91 ± 0.07 (23.37–24.0)
Peak frequency (kHz)	7.58 ± 0.57 (4.78–12.38)	8.92 ± 0.68 (5.91–13.03)
Delta frequency (kHz)	20.59 ± 0.25 (19.00–22.53)	21.22 ± 0.24 (19.84–22.30)
Aggregate entropy (bits)	6.45 ± 0.19 (4.73–7.28)	6.95 ± 0.12 (6.54–7.48)

**Table 3:** Results of the general linear models for repeated and non-repeated measures to test the effect of treatment (threat vs. capture), sex (female vs. male), and their interaction upon seven spectro-temporal characteristics of the hisses uttered by *Pristidactylus valeriae*. Data for the simple and with modulation patterns were pooled. Presented values are the F-statistics (*p*-value); statistically significant tests (*p* < 0.05) are shown in bold. *n* = sample size, df = degree of freedom.

	Repeated Measures (*n* = 8; df = 1,8)			Non-Repeated Measures (*n* = 7 Captured, 9 Threatened; 9 ♀, 7 ♂; df = 1,12)		
Variable	Treatment	Sex	Treatment × Sex	Treatment	Sex	Treatment × Sex
Duration (ms)	**8.092 (0.029)**	1.004 (0.355)	0.078 (0.789)	**5.531 (0.037)**	0.0000 (0.988)	0.273 (0.611)
Low Frequency (kHz)	4.604 (0.076)	0.997 (0.357)	2.111 (0.196)	0.472 (0.505)	0.733 (0.409)	0.633 (0.441)
Center frequency (kHz)	0.386 (0.557)	0.260 (0.628)	0.009 (0.926)	0.804 (0.387)	2.826 (0.119)	3.003 (0.109)
High frequency (kHz)	0.600 (0.470)	**7.600 (0.033)**	0.400 (0.546)	1.200 (0.295)	1.200 (0.295)	2.560 (0.136)
Peak frequency (kHz)	0.138 (0.723)	1.009 (0.354)	0.088 (0.777)	**5.117 (0.043)**	1.453 (0.251)	0.078 (0.785)
Delta frequency (kHz)	2.621 (0.157)	2.582 (0.159)	0.406 (0.548)	4.270 (0.061)	0.070 (0.793)	**7.330 (0.019)**
Aggregate entropy (bits)	4.530 (0.077)	4.586 (0.076)	0.281 (0.615)	4.316(0.060)	0.139 (0.716)	**5.920 (0.032)**

**Table 4:** Mean values of spectro-temporal variables of the hisses of three *Pristidactylus* species emitted by lizards threatened by a predator.

Species	Duration (ms)	Center Frequency	Delta Frequency	Reference
*P. scapulatus*	455 *	-	<4 kHz	Laspiur et al. [20]
*P. valeriae*	270	>8 kHz	>20 kHz	This study
*P. volcanensis*	429	<4 kHz	<4 kHz	Labra et al. [25]

* Average duration of the hisses recorded while animals attack or exhibit advertisement displays.

## Data Availability

The data set supporting the results will be available upon reasonable request.
